# Wire Arc Additive Manufacturing of AZ31 Magnesium Alloy: Grain Refinement by Adjusting Pulse Frequency

**DOI:** 10.3390/ma9100823

**Published:** 2016-10-09

**Authors:** Jing Guo, Yong Zhou, Changmeng Liu, Qianru Wu, Xianping Chen, Jiping Lu

**Affiliations:** 1 School of Mechanical Engineering, Beijing Institute of Technology, Beijing 100081, China; guojingcn@hotmail.com (J.G.); bitzhouy@bit.edu.cn (Y.Z.); qrwu@foxmail.com (Q.W.); jipinglu@bit.edu.cn (J.L.); 2 Beijing Institute of Astronautical Systems Engineering, Beijing 100076, China; chenxianping2000@gmail.com

**Keywords:** wire arc additive manufacturing, AZ31 magnesium alloy, grain, mechanical properties

## Abstract

Wire arc additive manufacturing (WAAM) offers a potential approach to fabricate large-scale magnesium alloy components with low cost and high efficiency, although this topic is yet to be reported in literature. In this study, WAAM is preliminarily applied to fabricate AZ31 magnesium. Fully dense AZ31 magnesium alloy components are successfully obtained. Meanwhile, to refine grains and obtain good mechanical properties, the effects of pulse frequency (1, 2, 5, 10, 100, and 500 Hz) on the macrostructure, microstructure and tensile properties are investigated. The results indicate that pulse frequency can result in the change of weld pool oscillations and cooling rate. This further leads to the change of the grain size, grain shape, as well as the tensile properties. Meanwhile, due to the resonance of the weld pool at 5 Hz and 10 Hz, the samples have poor geometry accuracy but contain finer equiaxed grains (21 μm) and exhibit higher ultimate tensile strength (260 MPa) and yield strength (102 MPa), which are similar to those of the forged AZ31 alloy. Moreover, the elongation of all samples is above 23%.

## 1. Introduction

As the lightest structural alloys, magnesium alloys have attracted great attention in recent years. They possess many excellent properties, such as high specific strength, good mechanic damping properties, electromagnetic shielding properties, castability, machining property, weldability, recyclability, biodegradability etc. [1,2,3,4]. Therefore, they have been widely used in aerospace, aircraft, automotive, electronics, and other industries [5,6]. For example, in the automotive industry, magnesium alloys play an important role in reducing weight and realizing energy saving and emission reduction by replacing steel and aluminum parts [7,8,9]. AZ31 magnesium alloy is one of the Mg–Al–Zn (AZ) alloys, and typically contains 2.5~3.5 wt % Al, 0.6~1.4 wt % Zn and 0.2~1.0 wt % Mn. At present, AZ31 magnesium alloy is the most widely used among all wrought magnesium alloys.

Currently, to realize further lightweight, developing monolithic components is an important trend for the application of magnesium alloy. However, the monolithic components generally exhibit large scale and complex shape. It is difficult to fabricate them using conventional forging and casting techniques [10,11,12]. Thus, new manufacturing methods are highly desired to produce monolithic magnesium alloy components.

Additive manufacturing (AM) is a technique that fabricates components layer upon layer by adding metallic materials vice conventional subtractive manufacturing [13,14,15]. It is a promising method for manufacturing complex and large scale monolithic components. During AM, a heat source (arc, electronic beam or laser) moves along a specific path and melts metal powder or wire to form the components [13]. In recent years, metal AM technology has been developed rapidly due to its ability of near-net shape fabrication of monolithic components and the possibility of reducing lead-time, energy consumption and cost [14,16,17,18,19]. Up to now, only a few researches on manufacturing pure magnesium [20,21], magnesium alloys [10,11] or the mixture of magnesium [22] using selective laser melting (SLM) have been reported. However, during SLM, most of laser energy is reflected owing to the low absorption of magnesium [23], which results in poor efficiency and increases the risk of the breakdown of the laser device. Besides, the fine powders of magnesium alloys for SLM are flammable and explosive.

Wire arc additive manufacturing (WAAM) is another choice of AM techniques. To fabricate parts or components, metal wire is fed at a constant speed and melted by arc onto a substrate or the previous deposition layers [24]. Compared with the power-based AM technique, WAAM has a much higher deposition rate, higher material usage efficiency and lower cost, which reveals the potential to fabricate large scale components [24,25,26,27,28]. Hence, WAAM has attracted great attention and has been reported in many papers to fabricate various materials, such as steel [29], nickel aluminum bronze alloy [30], titanium alloys [17,18,31], aluminum alloys [32,33] etc. However, to the best of our knowledge, studies focused on the WAAM of magnesium or its alloys have not been reported. Investigation of the WAAM of AZ31 magnesium alloy may provide an alternative method, allowing a broader application of magnesium and its alloys.

Hence, in this study, the WAAM of AZ31 magnesium alloy is investigated. Meanwhile, one of the main challenges for the widespread use of AM is to obtain fine grains and good mechanical properties. This study focuses on achieving this goal by adjusting pulse frequency. Thus, the influence of pulse frequency on macrostructure, microstructure and tensile properties of wire arc additive manufactured AZ31 magnesium alloy is disclosed in this paper.

## 2. Experimental Procedures

### 2.1. Experimental Setup and Manufacturing Process

**Figure 1:**
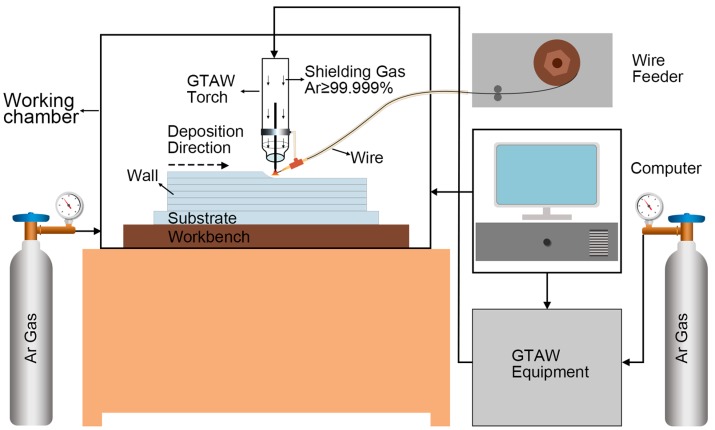
Schematic of the experimental setup developed for wire arc additive manufacturing.

Figure 1 schematically depicted the experimental setup developed for wire arc additive manufacturing. It was composed of gas tungsten arc welding (GTAW) equipment, a wire feeder, a computer, argon gas, a three-dimensional workbench and working chamber.

AZ31 wire with a diameter of 1.2 mm was used in this study. The rolled AZ31 plate with the size of 150 × 150 × 5 mm^3^ (length × width × height) was used as a substrate, which was mechanically cleaned and then fixed on the workbench before the deposition process.

To investigate the influence of pulse frequency, six wire arc additive manufactured walls of about 100 mm in length and 10~20 mm in height were manufactured by GTAW equipment (Make: Miller, Appleton, WI, USA; Model: Dynasty 350) with a WF-007A cool wire feeder (Make: WEILD, Guangzhou, China) in an argon atmosphere. Each wall was made up of eight layers. No preheating was conducted before the deposition process and the first layer was manufactured without a pulse current in order to obtain good shape. Other layers were manufactured with different pulse frequencies. There was about a one-minute period to cool down between depositing each layer. According to careful analysis of previous work about the GTAW of AZ31 magnesium alloy [7,34,35,36], and multiple trail experiments, optimized fabrication parameters were determined. Six different pulse frequencies were selected, and other fabrication parameters were kept constant. The main deposition parameters used in this study are shown in Table 1.

**Figure 2:**
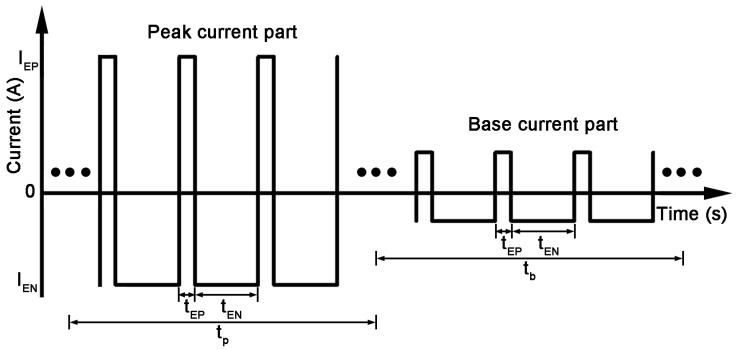
Schematic of the waveform used in this study.

The waveform used in this study was a combination of pulse and AC waveform as shown in Figure 2. The average current can be calculated by the following relation:

(1) $$I_{average}=\frac{\left(I_{EP}\times t_{EP}+I_{EN}\times t_{EN}\right)\times t_{p}+\left(I_{EP}\times t_{EP}+I_{EN}\times t_{EN}\right)\times k_{bp}\times t_{b}}{t_{p}+t_{b}}$$

where *I_average_* is the average current, *I_EP_* is the electrode positive, *I_EN_* is the electrode negative, *t_EP_* is the electrode positive duration, *t_EN_* is the electrode negative duration, *t_p_* is the peak current duration, *t_b_* is the base current duration and *k_bp_* is the base-to-peak current ratio. Furthermore, the average current in this study was 65 A.

### 2.2. Research Methodology

The influence of pulse frequency is analyzed from three aspects: macrostructure, microstructure and tensile properties. The height of the wire arc additive manufactured walls was measured and was divided by the total number of layers to obtain the average layer thickness. In consideration of the low geometry accuracy of walls, maximum width is chosen as another reference for geometric accuracy in this study.

**Figure 3:**
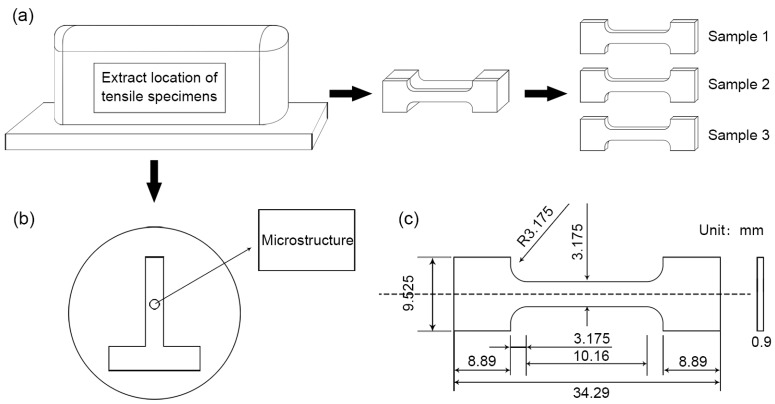
Sample preparation: (**a**) manufacturing procedure of tensile specimens; (**b**) cross sections for microstructure observations and (**c**) dimensions of tensile specimens.

Figure 3 shows the sample preparation procedures. Cross sections were cut for microstructure observations as shown in Figure 3b. The metallographic specimens were mounted, polished with SiC papers (180, 400, 600, 800, 1000, 1500, 2000 grit), and then electrolytic polished in ethanol 90% and perchloric acid 10% for about one minute at −30 °C and 15 V. After that, they were etched in a mixture of 5 g picric acid, 5 mL acetic acid, 10 mL diluted water and 100 mL ethanol. The microstructures of samples were characterized by optical microscopy (Make: Leica, Wetzlar, Germany; Model: Leica DM4000M). The first (bottom) and last (top) several layers have different microstructures due to the incomplete thermal cycles [37]. To avoid this influence, the middle of the walls was selected to reveal the microstructures and analyze the tensile properties.

**Figure 4:**
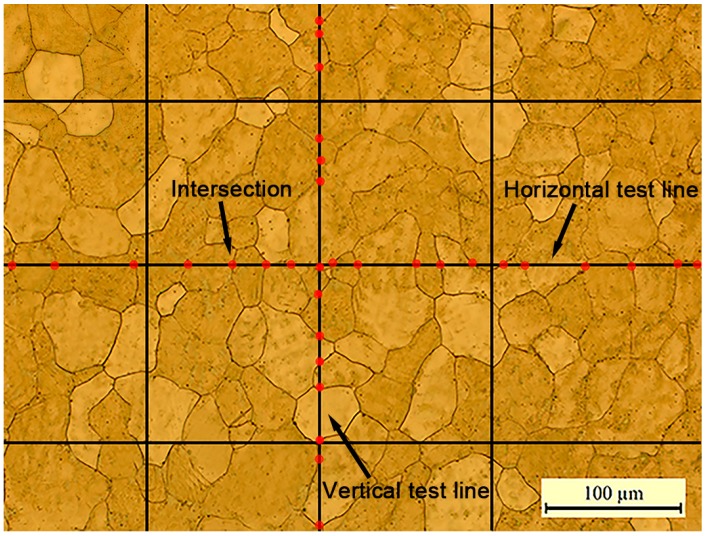
Sketch of the line intercept method.

In order to quantitatively investigate the effect of pulse frequency on microstructures, the line intercept method is adopted to measure the grain size and the grain aspect ratio. The details are described in Figure 4. Firstly, three test lines were evenly placed in the microstructures along the horizontal and vertical directions. Secondly, the interceptions were counted. An intersection point represents that one test line cuts a grain boundary. The counting number is set to 1 for each intersection, 1 for each tangential intersection, 0.5 when one end of the test line ends exactly on a grain boundary, and 1.5 when the intersection occurs at a triple point. Thirdly, the grain size is obtained by,

(2) $$d=\frac{l}{N}$$

where *d* is grain size, *l* is the length of the test line and *N* is the number of the intersections. Finally, at least three different microstructures of every sample were measured. The grain size was calculated by averaging the measured values. In this way, grain size along the horizontal and vertical direction was obtained respectively. Different grain sizes along different directions were observed in this experiment. Therefore, the grain aspect ratio, defined as the grain size along the vertical direction divided by the grain size along the horizontal direction, is analyzed, which to some extent reflects the anisotropic mechanical properties.

Figure 3a presents the manufacturing procedure of the tensile specimens. These specimens were cut from the middle parts of the walls along the longitudinal direction and then machined to the required dimensions by electric discharge wire cutting. The tensile properties were evaluated by an electronic universal material testing machine (Make: Instron, Darmstadt, Germany; Model: Instron 5966) at a strain rate of 9.8 × 10^−4^ s^−1^ at room temperature. The tensile properties were calculated by averaging the measured data of three tensile samples. The dimensions of tensile specimens are shown in Figure 3c. Besides, the fracture surfaces of the tensile specimens were characterized by a scanning electron microscope (SEM) (Make: JEOL, Tokyo, Japan; Model: JSM-6610LV).

## 3. Results and Discussion

### 3.1. Macrostructure

**Figure 5:**
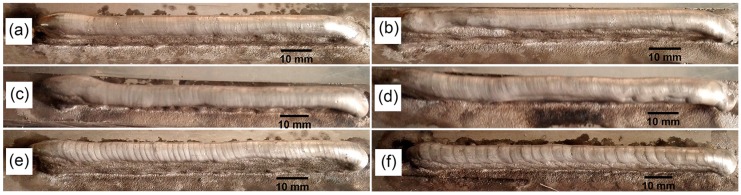
Surface morphologies of the samples deposited by different pulse frequencies: (**a**) 500 Hz; (**b**) 100 Hz; (**c**) 10 Hz; (**d**) 5 Hz; (**e**) 2 Hz and (**f**) 1 Hz.

Figure 5 presents the surface morphologies of the additively manufactured samples fabricated at different pulse frequencies. Coarse periodic weld ripples are observed at the surface of the walls deposited by low pulse frequency as shown in Figure 5e,f. With the increase of the pulse frequency, periodic weld ripples become finer. Moreover, at high pulse frequency, there are so many weld ripples that they seem to become continuous. The wall surface is smoother as shown in Figure 5a,b. It can be seen that pulse frequency has a significant effect on the surface morphologies. Weld ripples are indications of solidified droplets. During the welding process, the wire is melted at the peak current period and the molten droplet is adhered to the wire due to the surface tension at the base current period. When the base current turns into the peak current, previous molten droplet drops on the substrate or the previous deposition layers. Hence, at a low pulse frequency, there is more time for the droplet to become bigger, resulting in weld ripples being coarser and less than those at high frequency. Besides, the number of weld ripples indicates the number of weld pool oscillations. As pulse frequency increases, the molten pool is stirred more intensely.

**Figure 6:**
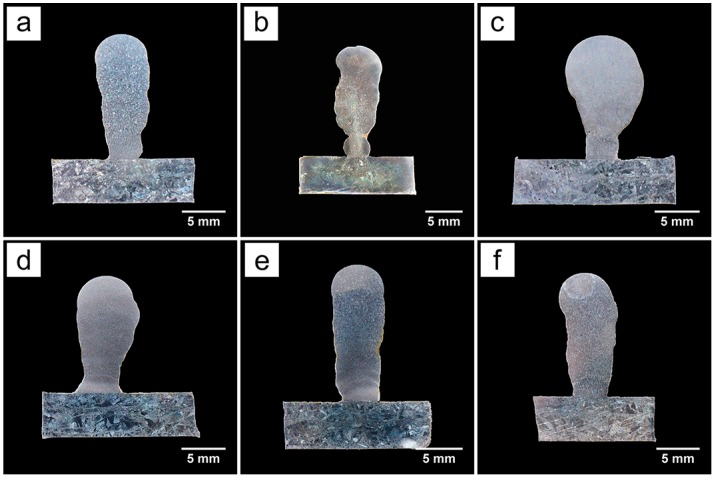
Cross sections of the samples deposited by different pulse frequencies: (**a**) 500 Hz; (**b**) 100 Hz; (**c**) 10 Hz; (**d**) 5 Hz; (**e**) 2 Hz and (**f**) 1 Hz.

**Figure 7:**
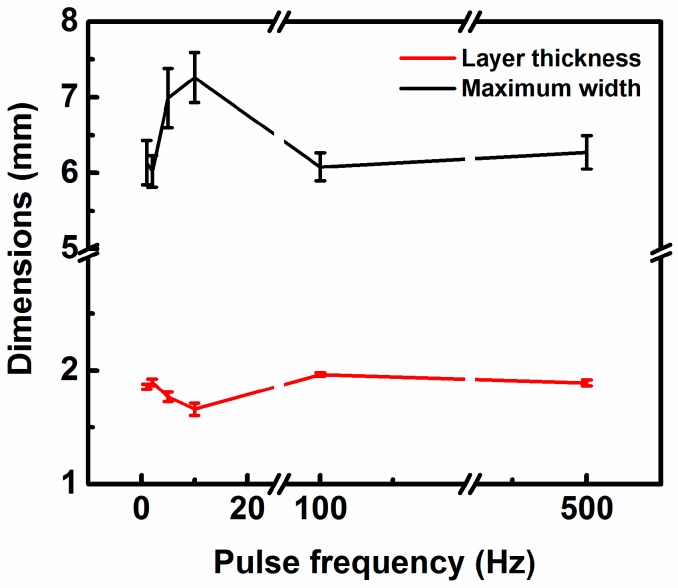
Effect of pulse frequency on the geometry of deposited layers.

Figure 6 displays the cross sections of the samples deposited by different pulse frequencies. At a lower pulse frequency (F < 5), a multilayer sample is deposited with few changes in its width from the substrate to the top as shown in Figure 6e,f. At the frequencies of 5 Hz and 10 Hz, a multilayer sample is deposited with its width becoming larger from the substrate to the top as shown in Figure 6c,d. At a higher pulse frequency (F > 10), a multilayer sample is deposited as its width is smaller than that of samples deposited by 5 Hz and 10 Hz and changes slightly from the substrate to the top as shown in Figure 6a,b. Figure 7 quantitatively describes the effect of the pulse frequency on the geometry of deposited layers. It can be seen that as the pulse frequency increases, the layer thickness decreases firstly and then increases and remains stable, while the maximum width increases firstly and then decreases to a certain value. The sample fabricated at the frequency of 10 Hz has the smallest layer thickness (1.7 mm) and the largest maximum width (7.3 mm).

Within the bounds of statistical variation, the geometry of the 1, 2, 100, and 500 Hz samples are all virtually identical. The only ones that are statistically different are the 5 Hz and 10 Hz samples. The geometry of deposited layers is determined by the shape of the weld pool. Pulse current promotes the fluid flow and agitation of the weld pool [24], and with the increase of pulse frequency, the molten pool is stirred more intensely as mentioned above. It is likely that a frequency of 5–10 Hz causes the weld pool to oscillate at its natural frequency. This causes the odd shaped deposits with these frequencies.

### 3.2. Microstructure

**Figure 8:**
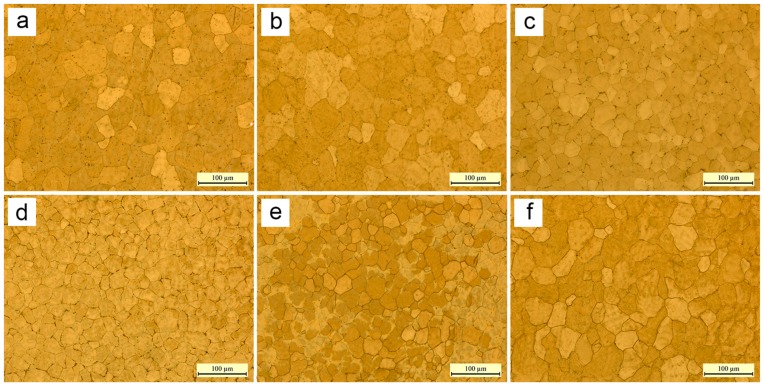
Microstructures of the samples deposited by different pulse frequencies: (**a**) 500 Hz; (**b**) 100 Hz; (**c**) 10 Hz; (**d**) 5 Hz; (**e**) 2 Hz and (**f**) 1 Hz.

Figure 8 exhibits the microstructures of the samples deposited by different pulse frequencies. No pore defect is observed in these microstructure images and fully dense AZ31 magnesium alloy components are obtained. It is noted that there are huge differences between these microstructures in terms of grain. Table 2 summarizes the grain size of samples fabricated by different pulse frequencies. With the enhancement of pulse frequency, grain size decreases firstly and then increases. The microstructures of samples fabricated by 5 Hz and 10 Hz are fairly fine and uniform, whose grain size is 21 μm as shown in Figure 8c,d. Coarse grains can be observed in Figure 8a,b,f. The grain size of the sample fabricated by 100 Hz is even up to 39 μm.

Besides, Table 2 summarizes the grain aspect ratio of samples fabricated by different pulse frequencies. The grain aspect ratio reflects whether there are differences in grain size along the horizontal and vertical direction, which indicate anisotropic mechanical properties. The grain aspect ratio of the samples fabricated at 5 Hz and 10 Hz is close to 1, which means the same grain size along the horizontal and vertical direction as shown in Figure 8c,d and indicates isotropic mechanical properties. The grain aspect ratio of the 1, 2, 100, and 500 Hz samples is slightly larger than 1, which indicates slightly anisotropic mechanical properties.

Apparently, pulse frequency has an appreciable influence on microstructure, especially grain size and grain aspect ratio. There are mainly two factors of pulse frequency contributing to the grain growing behavior.

**Figure 9:**
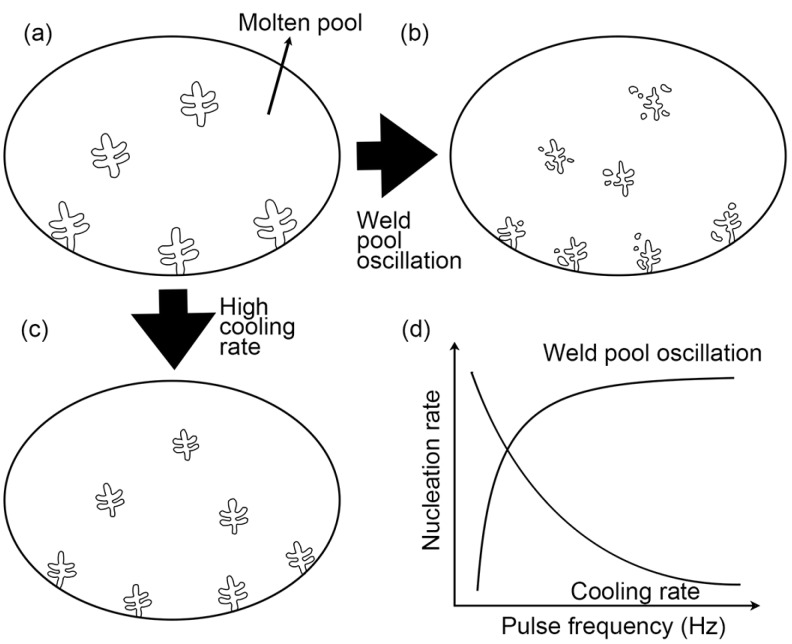
Schematic diagram of the effect of pulse frequency on nucleation: (**a**) conventional nucleation without pulse current; (**b**) nucleation under weld pool oscillations; (**c**) nucleation under high cooling rate and (**d**) nucleation rate curves.

Firstly, pulse current can cause the stirring of the molten pool, leading to refining grains. Increased pulse current results in an increased plasma momentum and increases the value of the electromagnetic force. Plasma momentum creates arc pressure and shearing force on the surface of the weld pool. Electromagnetic force inside the weld pool pushes the fluid radially inwards and then down the axis [34]. This can produce enough weld pool oscillations and cause dendrite fragmentation to provide more heterogeneous nucleation sites so as to refine the grain size [13] as shown in Figure 9b. As the pulse frequency grows, weld pool oscillations increase, which is consistent with the phenomenon of the weld ripples, and the grain refinement effect is increased.

Secondly, the pulse current can cause the high cooling rate of the molten pool, resulting in refining grains. The peak current is mainly used to input heat to melt the metal wire while the base current is much smaller than the peak current and is used to maintain the arc. During the base current, the heat input was suddenly reduced and therefore the cooling rate of the molten pool is high causing heterogeneous nucleation to refine grains [38,39] as shown in Figure 9c. When pulse frequency changes, total heat input remains constant. With the increase of pulse frequency, arc pressure is becoming higher, resulting in a more constricted and longer arc, so that arc is large with a conical shape at first, and then becomes smaller and finally even appears to be steady in a near cylindrical shape. Meanwhile, as the pulse frequency increases, the duration of the base current becomes shorter and the time for heat dissipation is reduced. In a word, higher pulse frequency results in a larger effective heat input, higher temperature and lower cooling rate [34].

In the case of the two reasons above, there exists an optimum pulse frequency of maximum grain refinement due to both more weld pool oscillations and a higher cooling rate of the molten pool, leading to more heterogeneous nucleation sites as shown in Figure 9d. In this study, the optimum pulse frequency is found to be 5 Hz and 10 Hz. The samples fabricated at 5 Hz and 10 Hz contain finer equiaxed grains (21 μm) whose grain aspect ratio is close to 1 at the same time. Meanwhile, the resonance of the weld pool at these frequencies, as mentioned previously, greatly promotes grain refinement.

### 3.3. Tensile Properties

Tensile testing was conducted to discover the effect of pulse frequency on mechanical properties. Table 3 summarizes the tensile properties of the samples fabricated by different pulse frequencies, including ultimate tensile strength (UTS), yield strength (YS) and elongation (EL). The tensile properties change significantly with the variation of pulse frequency (UTS is from 221 to 263 MPa and YS is from 79 to 104 MPa). It should be noted that the samples fabricated at 5 Hz and 10 Hz exhibit higher UTS (260 MPa) and YS (102 MPa), which are similar to those of the forged AZ31 alloy whose UTS is 234 MPa and YS is 131 MPa according to ASTM standard B91-12 [40]. Moreover, the elongation of all samples is above 23%, which indicates good plastic property.

**Figure 10:**
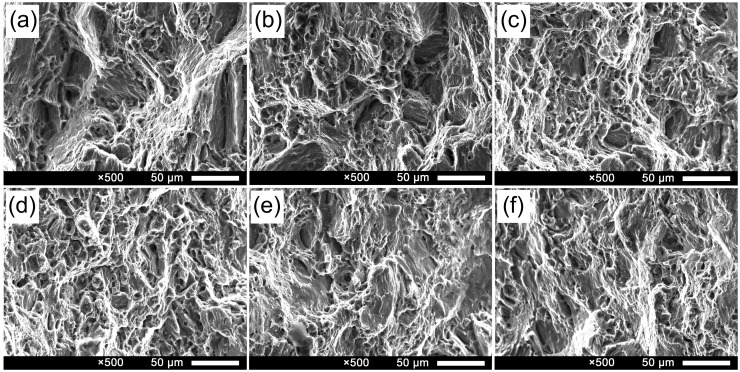
SEM fractographs of tensile samples deposited by different pulse frequencies: (**a**) 500 Hz; (**b**) 100 Hz; (**c**) 10 Hz; (**d**) 5 Hz; (**e**) 2 Hz and (**f**) 1 Hz.

Figure 10 shows the SEM fractographs of tensile samples deposited by different pulse frequencies. It is clear that all fracture surfaces present a dimple rupture. Compared with others, the fracture surfaces of the tensile specimens manufactured at 5 Hz and 10 Hz contain a lot of much finer dimples which indicate relatively higher tensile strength. The fractographs are consistent with tensile properties as well as the microstructure.

The samples fabricated at 5 Hz and 10 Hz contain finer equiaxed grains with no pore defect and achieve the maximum tensile strength in comparison to others. Moreover, the larger the grain size is, the worse the tensile strength becomes. Many researchers have studied the relationship between mechanical properties and microstructure. The mechanical property of the alloy is directly affected by the average grain size according to the Hall–Petch equation [41,42]. Therefore, the formation of finer grains of the samples contributes to higher tensile strength and coarser grains easily lead to poor tensile properties. Grain size depends on the process parameters and pulse frequency can refine grains and enhance tensile properties by controlling weld pool oscillations and the cooling rate of the molten pool.

It can be concluded that pulse frequency has a remarkable influence on microstructure and mechanical properties of wire arc additive manufactured AZ31 magnesium alloy. Fine microstructure and good mechanical properties can be obtained by adjusting pulse frequency. The results indicate that optimum pulse frequency is found to be 5 Hz and 10 Hz, which leads to finer equiaxed grains and better mechanical properties compared with other pulse frequencies. Although the shape of samples fabricated at 5 Hz and 10 Hz is poor, good geometry accuracy can be reached by adjusting the relative manufacturing process. The arc current can control the geometry of deposited layers [32] and reducing the arc current is often used to obtain good geometry accuracy during deposition [17,18].

## 4. Conclusions

Wire arc additive manufacturing of AZ31 magnesium alloy has been carried out in this investigation. The effects of pulse frequency on macrostructure, microstructure and tensile properties of the arc additive manufactured samples are analyzed. From this investigation, the important conclusions are as follows:

(1) Fully dense AZ31 magnesium alloy components are successfully fabricated using WAAM. WAMM has the potential to manufacture magnesium alloys components.
(2) Pulse frequency has a significant influence on macrostructure, microstructure and tensile properties of the wire arc additive manufactured samples. The shape of samples fabricated at 5 Hz and 10 Hz is odd due to the weld pool oscillating at its natural frequency. As the pulse frequency grows, weld pool oscillations increase and the cooling rate decreases leading to the fact that grain size decreases firstly and then increases, meanwhile, the grain aspect ratio is close to 1 firstly and then away from 1. Grain refinement reaches the maximum at 5–10 Hz, owing to resonance of the weld pool at these frequencies as mentioned previously. Correspondingly, tensile strength increases firstly and then decreases.
(3) Fine grains and good tensile properties can be obtained at a certain pulse frequency. The samples fabricated at 5 Hz and 10 Hz contain finer equiaxed grains (21 μm) and exhibit higher ultimate tensile strength (260 MPa) and yield strength (102 MPa), which are similar to those of the forged AZ31 alloy. Moreover, the elongation of all samples is above 23%.

## Figures and Tables

**Table 1:** Deposition parameters used in this study.

Deposition Parameters	Values
Type of welding current	alternating current (AC)
Alternating current frequency	400 Hz
Electrode positive (EP)	136 A
Electrode negative (EN)	91 A
Electrode negative ratio	80%
Pulse frequency	1, 2, 5, 10, 100 and 500 Hz
Peak time ratio	50%
Base-to-peak current ratio	30%
Wire feed rate	2 m/min
Deposition speed	200 mm/min
Argon	99.999% purity
Shield gas flow rate	20 L/min
Arc length	3 mm
Tungsten electrode diameter	2.4 mm

**Table 2:** Grain size and grain aspect ratio of the samples fabricated by different pulse frequencies.

Pulse Frequency (Hz)	Grain Size (μm)	Grain Aspect Ratio
1	31 ± 4	1.09 ± 0.04
2	23 ± 1	1.15 ± 0.06
5	21 ± 1	1.02 ± 0.12
10	21 ± 1	0.98 ± 0.11
100	39 ± 4	1.22 ± 0.21
500	37 ± 3	1.14 ± 0.10

**Table 3:** Tensile properties of the samples fabricated by different pulse frequencies.

Pulse Frequency (Hz)	UTS (MPa)	YS (MPa)	EL (%)
1	229 ± 3	90 ± 1	26.3 ± 0.3
2	232 ± 1	89 ± 1	26.7 ± 1.2
5	258 ± 5	100 ± 3	25.6 ± 3.1
10	263 ± 5	104 ± 5	23.0 ± 3.7
100	221 ± 11	82 ± 6	23.4 ± 5.8
500	231 ± 5	79 ± 2	27.3 ± 0.3
